# Enhanced air pollution via aerosol-boundary layer feedback in China

**DOI:** 10.1038/srep18998

**Published:** 2016-01-12

**Authors:** T. Petäjä, L. Järvi, V.-M. Kerminen, A.J. Ding, J.N. Sun, W. Nie, J. Kujansuu, A. Virkkula, X. Yang, C.B. Fu, S. Zilitinkevich, M. Kulmala

**Affiliations:** 1 Department of Physics, University of Helsinki, Finland; 2 Joint International Research Laboratory of Atmospheric and Earth System Sciences & School of Atmospheric Sciences, Nanjing University, China; 3 Finnish Meteorological Institute, Helsinki, Finland; 4 University of Nizhny Novgorod, Nizhny Novgorod, Russia; 5 Moscow State University, Moscow, Russia; 6 Institute of Geography, Russian Academy of Sciences, Moscow, Russia

## Abstract

Severe air pollution episodes have been frequent in China during the recent years. While high emissions are the primary reason for increasing pollutant concentrations, the ultimate cause for the most severe pollution episodes has remained unclear. Here we show that a high concentration of particulate matter (PM) will enhance the stability of an urban boundary layer, which in turn decreases the boundary layer height and consequently cause further increases in PM concentrations. We estimate the strength of this positive feedback mechanism by combining a new theoretical framework with ambient observations. We show that the feedback remains moderate at fine PM concentrations lower than about 200 μg m^−3^, but that it becomes increasingly effective at higher PM loadings resulting from the combined effect of high surface PM emissions and massive secondary PM production within the boundary layer. Our analysis explains why air pollution episodes are particularly serious and severe in megacities and during the days when synoptic weather conditions stay constant.

Economic growth has been very fast in China during the last decades^1^. The rapid industrialization associated with this growth and high pollutant emissions^2^,^3^,^4^,^5^,^6^ has come with a price, as the strong need for resources in terms of both energy and raw materials has caused serious environmental problems^7^,^8^,^9^,^10^,^11^. As an example, poor air quality^12^,^13^ in China has been estimated to cause 1.27 million premature deaths annually^14^. Even higher estimates have been given by the most recent World Health Organization (WHO) assessment which attributes 7 million premature deaths world-wide in 2012 to air pollution, making it the single largest cause of death in the world^15^. The adverse health effects are caused mainly by aerosol particles suspended in the near surface air, with additional contribution coming from trace gases like ozone^16^. Concentrations of aerosol particles have increased considerably over China during the past decades^17^, and the most populated regions in there have experienced frequent and severe air pollution episodes during the recent years^7^,^8^,^9^,^10^,^11^,^12^,^13^,^18^,^19^,^20^.

Poor air quality is the result of several factors tied with pollutant emissions, atmospheric transport, atmospheric chemistry and meteorological conditions. Emissions of primary particles and aerosol precursors have experienced substantial chances in China during the last 2–3 decades, the current emissions levels being substantially higher than those in the past^2^,^3^,^4^,^5^,^6^. High aerosol concentrations tend to occur when meteorological conditions favor the accumulation of primary and secondary pollutants in the air near the surface, yet the ultimate cause for the most severe air pollution episodes in China has remained unsolved^10^,^12^,^13^,^18^,^19^,^20^,^21^,^22^. The key player in this respect is the boundary layer (BL), which is the lowest atmospheric layer immediately affected by the Earth’s surface. In weather and climate systems, the BL acts as a strongly turbulent buffer coupling the surface with the free troposphere. In contrast to the BL, the bulk of the atmosphere is weakly turbulent because of the strongly stable stratification. In a sense, the upper boundary of the BL makes a lid that essentially weakens the BL-free troposphere exchange. In particular, variations in temperature, driven by the diurnal course of solar irradiation at the surface, are strongly pronounced close to the surface and decay non-linearly with the intensity of turbulence towards the upper boundary of the BL. Similarly, aerosols, dust, gases and any other admixtures released from ground sources are to a large extent blocked within the BL. The BL depth varies from a few dozen meters to a few kilometers and it represents an important parameter controlling heavy air-pollution episodes, extreme colds or heat waves, and local consequences of the dispersal of ground level aerosol particles^23^.

We demonstrate here that the anthropogenic particulate pollution generates a strong lid on the top of the BL, hindering turbulent mixing of pollutants from the surface to higher up. While aerosol particles are known to influence the boundary layer stability^24^,^25^,^26^, general understanding of this phenomenon in heavily-polluted environments, like those encountered in China, has remained very low^12^,^20^,^27^,^28^. Here we combine a theoretical analysis and atmospheric observations to show that aerosol particles increase the BL stability and cause any subsequent emissions to remain in a lower BL, giving rise to a positive feedback on pollutant concentrations that reduces the mixing height even further (Fig. 1). We estimate the strength of this feedback by linking the surface energy balance with aerosol concentrations and BL height using real atmospheric data. This feedback mechanism emerges only at high pollutant concentration typical for the most populated regions in China, but it is expected to be operative in all heavily-polluted BLs of world’s megacities.

A generalized surface energy balance in urban settings can be described as follows^29^,^30^^, SI^: the surface is heated by incoming solar radiation from the sun and long-wave radiation from the atmosphere above. Depending on the surface albedo, a fraction of the incoming solar radiation is reflected back to the atmosphere. Furthermore, the surface emits long-wave radiation to the atmosphere depending on the surface temperature. The resulting net energy is distributed between the turbulent sensible and latent heat components along with the net heat advection and heat storage terms. The intensity of turbulent mixing is governed by two factors: the wind shear that mechanically generates turbulence and the stratification of the boundary layer. The turbulent mixing is tied to the boundary layer height which determines the level up to which surface emissions will be distributed. Under unstable conditions, the stratification enhances turbulent mixing and makes the BL height to increase, whereas under stable conditions the stratification weakens the turbulence and reduces the BL depth.

The urban BL is usually unstably stratified^31^,^32^,^33^, and therefore deep and strongly turbulent, which yields effective vertical dilution and consequently removal of pollutants from the surface. We declare that this recognized conclusion holds only true for clean or for, at most, moderately polluted air. BLs typical for large Chinese cities and other megacities may exhibit dramatically different properties, such as comparatively low BL depth and extremely high level of air pollution^10^,^11^,^22^, especially during cloud-free days. We explain this paradox as a result of an unaccounted feedback between the aerosol mass concentration and the static stability of the BL. Suppose that the BL becomes heavily polluted due to the combined effect of aerosol emissions and secondary formation of particulate matter. As a result, the optical depth of the BL (and entire atmospheric column) increases, so the magnitude of short-wave radiation reaching the surface decreases, altering the energy balance (Eq. S1). A major share of the incoming solar radiation is absorbed by the heavily polluted layer, while some of it is scattered into all directions. The absorption changes the vertical temperature profile, heating the upper part of the boundary layer and leading to an increased stability^25^,^26^,^27^,^34^, which in turn reduces turbulence and mixing. Overall, this chain of processes reduces the atmospheric boundary layer height. According to our theoretical analysis (See SI), the ratio of the boundary layer height between the polluted and non-polluted conditions scales as the square root of vertical turbulent fluxes, provided that the synoptic-scale meteorological conditions remain the same.

A direct consequence of the lowered boundary layer height is an increase in aerosol concentrations as emissions dilute to a smaller volume. In other words, there is a negative feedback between the surface PM concentration and convective BL height via the vertical turbulent heat flux at the surface (henceforth referred to as “the surface heat flux”). In order to quantify this feedback, we combined our theoretical analysis with atmospheric observational data from Station for Observing Regional Processes of the Earth System (SORPES^35^), in Nanjing of the Yangtze River Delta, China.

In order to illustrate the above feedback mechanisms, a set of four days from May 9 to 13 2013 of atmospheric data from the SORPES station was chosen (Fig. 2). The instrumentation and associated data are described in the SI. During this time, aerosol mass concentration varied between 50 and 120 μg m^−3^, out of which approximately 10% was light-absorbing matter, black carbon. The down-welling short wave radiation (K_down_) was the lowest when the fine particle mass concentration was the highest, whereas the horizontal wind velocity remained quite constant during the whole period, indicating relatively constant synoptic-scale weather conditions. The boundary layer height during this period was at its lowest during the day having the highest fine particle mass concentration, qualitatively supporting our hypothesis on the feedback between the aerosol mass concentration and boundary layer height.

With a longer time data set (8 months of data presenting both summer and winter conditions in SORPES), we explored the relation between aerosol mass concentrations and radiation components. The data used represent hourly averages from non-rainy periods between 10:00 and 14:00. The maximum aerosol mass concentration during the observed period was about 300 μg m^−3^ during short episodes in December 2014. We normalized the components with theoretical short wave solar radiation at the top of the atmosphere (K_top_) to remove the seasonality and time of day effects. The surface observations showed that K_down_ and long-wave radiation as well as net radiation in all wave-lengths correlated negatively with the measured aerosol mass concentration (Supplementary Fig. A1). As an overall effect, the observed vertical turbulent flux (F_b_) showed a negative correlation with aerosol mass and black carbon concentrations, the correlation being somewhat stronger for the mass concentration (Fig. 3, SI Table A1 and SI Fig. A1 and A2). Although this correlation does not confirm causality, we argue that there is a cause-effect relationship between the increased concentration of aerosol particles, vertical turbulent flux and boundary layer height, based on our theoretical understanding.

The timescale in our observations is 1 h, whereas the boundary layer mixing time is on the order of 20−30 minutes. Therefore, the observed reduction in the vertical turbulent flux as with an increasing aerosol mass concentration in our analysis is the net effect of the whole feedback depicted in Fig. 1. We divided the data according to the ambient relative humidity (RH). The weaker dependency of the BL height reduction associated with a higher RH is presumably due to aerosol water uptake, which reduces the magnitude of the turbulent vertical flux (F_b_) already at low mass loadings (Fig. 3). In Nanjing, high humidities are usually associated with low turbulent fluxes, and consequently with low boundary layer heights. We ascribe this dependency to the aerosol hygroscopicity^36^. The fitting results show that the boundary layer height reduces to half of the original height at particle mass concentrations slightly above 200 μg m^−3^.

The fitting in Fig. 4, connecting the aerosol mass concentration to the reduction of boundary layer height, allows us to calculate the strength of the feedback mechanism. Consider a BL with a certain PM concentration and subsequent small increase in this concentration due to either surface PM emissions or secondary aerosol formation inside the BL. Assuming that the concentration increase occurs during conditions when horizontal advection and vertical entrainment to free troposphere remain constant, the net effect is that any further PM emission (or production) will be distributed into a shallower boundary layer, increasing the concentration further. The net PM concentration increase (ΔPM’) is therefore larger than if the same amount of PM would had been emitted to, or produced within, the original BL (ΔPM). The amplifying effect of this positive feedback (ΔPM’/ΔPM) depends on both the PM loading and rate of its increase. By assuming an initial fine PM concentration of 100, 200 or 250 μg m^−3^, the corresponding strength of the feedback is about 1, 2 and 5% for ΔPM < 10 μg m^−3^, about 3, 5 and 12% for ΔPM of 20 μg m^−3^ and 6, 13 and > 50% for ΔPM of 40 μg m^−3^. These values are in line with recent model simulations^20^,^21^. Under extremely polluted conditions (fine PM > 250–300 μg m^−3^), aerosol particles allow very little solar radiation to reach the surface, so that the boundary layer almost completely collapses even in a case that the majority of the emissions are dispersed above the polluted boundary layer. The only component providing vertical turbulence in such case is anthropogenic heating. This type of a situation remains until a change in synoptic weather conditions will provide fresh air from outside the megacity.

Our results show that aerosol-boundary layer feedback acts as a plausible explanation for the most severe haze episodes observed in the most populated regions of China. Based on our theoretical analysis supported with experimental data, we formed a new concept where the hindrance of mixing caused by the aerosol particles is directly apparent. Our analysis connects the atmospheric particulate matter to a reduction in the vertical turbulent mixing. This leads to a decrease of a boundary layer height, into which the air pollution from the surface is mixed. Taking this into an extreme, in a quite possible case of “supercritical” pollution, aerosol particles reduce the solar radiation at the surface to such a low level that the BL static stability would change from unstable to stable. This would cause the decay of turbulence and drop down of the BL height entailed by catastrophic increase in aerosol concentration. In a megacity, anthropogenic heating is a rather weak protection against such catastrophe. More generally, as pollutant concentrations increase due to increased emissions or unfavorable meteorological conditions, the net effect is that the boundary layer decreases further causing even poorer air quality. The feedback mechanism discussed here reduces the availability of solar energy for electricity in the megacities. Furthermore, existing models fail to forecast regional weather during extremely high episodes in China^19^,^27^, since the mechanism described here is not included in a proper way in models. Therefore, it is crucial to include our feedback-loop in air quality and weather forecasts and as part of an early-warning system for extreme air quality episodes.

## Methods

The Station for Observing Regional Processes of the Earth System (SORPES^35^) is a latest developed observation platform by Nanjing University (NJU) in collaboration with the University of Helsinki. The station is located in NJU at Xianlin Campus (N 32°7′14″, E 118°57′10″), in about 20 km east from the downtown Nanjing.

Measurements of trace gases, aerosols, and relevant meteorological parameters began in the summer of 2011. Most of the instruments are housed on the top floor of a laboratory building, which sits on the top of a hill about 40 m above the ground level. PM_2.5_ mass concentrations were measured using an on-line mass analyzer (Thermo SHARP-5030). The aerosol mass analyzer was operated under dry conditions, with a 1 m long DHS heater settled up to keep the RH of samples no larger than 35%. Black carbon was measured using a 7-wavelength Aethalometer (AE31) from MAGEE Scientific. Meteorological measurements have been available at the site with Automatic Weather Station (AG1000, Campbell Scientific Inc.). The three-dimensional wind speed fluctuation and virtual temperature were measured with an ultrasonic anemometer (CSAT3, Campbell Scientific Inc.), water vapor with EC150 (Campbell Scientific Inc.) and the data were collected and stored by a data logger (CR5000, Campbell Scientific Inc.) with a sampling frequency of 10 Hz. The framework for boundary layer development is presented in^37^. The theoretical derivation of the connection between the boundary layer height and aerosol particulate mass is presented in Supplementary Material.

## Additional Information

**How to cite this article**: Petäjä, T. *et al.* Enhanced air pollution via aerosol-boundary layer feedback in China. *Sci. Rep.* **6**, 18998; doi: 10.1038/srep18998 (2016).

## Supplementary Material

Supplementary Information

## Figures and Tables

**Figure 1:**
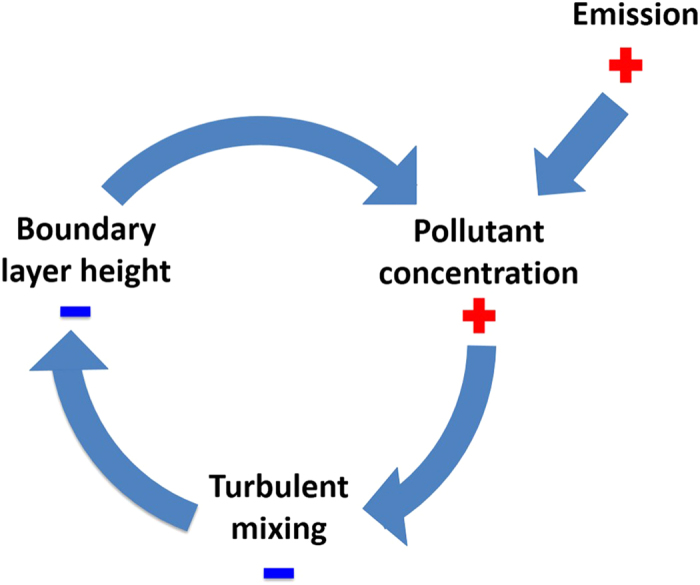
A schematic figure of the feedback mechanism initiated by the increased aerosol concentration in the boundary layer leading to lower boundary layer height and hence elevated aerosol concentrations.

**Figure 2:**
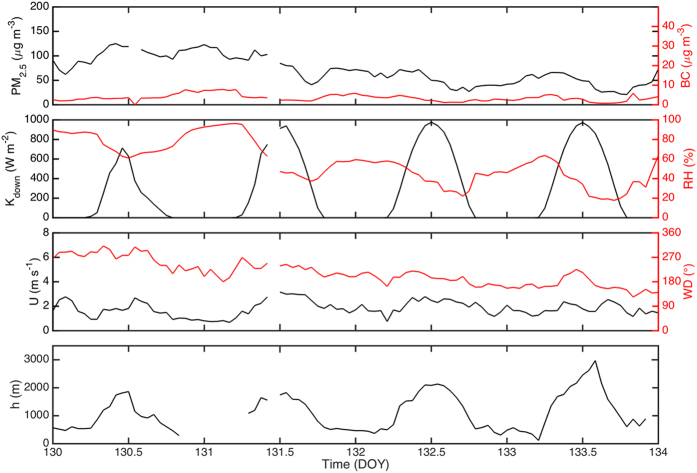
Time series of (**a**) fine aerosol particles (PM_2.5_) and black carbon (BC) mass concentrations, (**b**) solar radiation (K_down_) and relative humidity (RH), (**c**) wind speed (*U*) and direction (WD) and (**d**) the boundary layer height (h).

**Figure 3:**
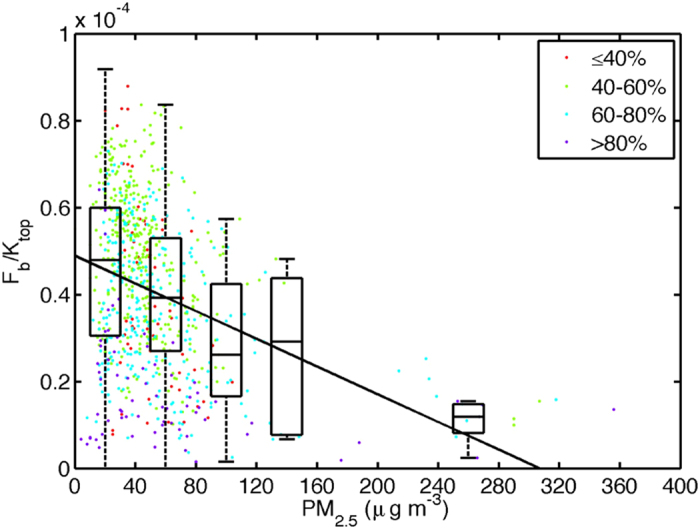
Observed dependency of the ratio between the turbulent vertical flux (F_b_) and the solar radiation at the top of the atmosphere (K_top_) as a function on observed particulate mass (PM_2.5_) concentration. The fitting includes all the data points. To guide the eye, the PM_2.5_ data is binned in five batches where the median is shown as a line whereas the outer boundaries of the boxes represent 25 and 75 quartiles and the dashed lines present interquartile range (IQR). Dashed vertical lines represent 5 and 95 percentile ranges in each bin. Only daytime conditions between 10:00 and 14:00 local time from non-rainy periods are considered. Atmospheric RH during the measurements is indicated with the color of the data point. The dependency of the other radiation components are presented on PM2.5 is presented in Supplementary Fig. A1 and on BC concentration in Supplementary Fig. A2.

**Figure 4:**
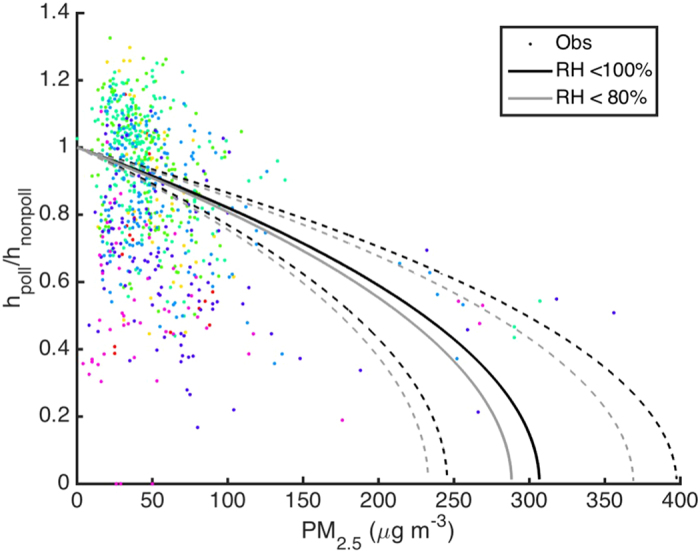
The anticipated change in the ratio of polluted to non-polluted boundary layer heights separately for all the available data and for relative humidity below 80%. The points present observational data. The color of the data points describe relative humidity with same classification as in Fig. 3. The solid lines present the fitted dependency and the dashed lines show the sensitivity of the results based on estimated fitting errors.
