# Supplementary material for: Enhanced air pollution via aerosol-boundary layer feedback in China
Source: Sci Rep. 2016 Jan 12;6:18998. doi: 10.1038/srep18998 (PMC4709519; doi:10.1038/srep18998)
Supplement: Supplementary Information [file srep18998-s1.docx]

Supplementary Materials and Methods for

Enhanced air pollution via aerosol-boundary layer feedback in China

T. Petäjä^1*^, L. Järvi^1^, V.-M. Kerminen^1^, A. Ding^2*^, J. Sun^2^, W. Nie^1,2^, J. Kujansuu^1^, A. Virkkula^2,3^, X. Yang^2^, C. Fu^2^, S. Zilitinkevich^1,3,4^ and M. Kulmala^1*^

^1^Department of Physics, University of Helsinki, Finland

^2^Institute for Climate and Global Change Research & School of Atmospheric Sciences, Nanjing

University, China

^3^Finnish Meteorological Institute, Helsinki, Finland

^4^University of Nizhny Novgorod, Russia

*Correspondence to: tuukka.petaja@helsinki.fi, dingaj@nju.edu.cn, markku.kulmala@helsinki.fi

**A1 Theoretical derivation of boundary layer height in polluted urban convective boundary layer**

**A1.1 Energy balance**

The energy balance at the Earth surface reads (in W m^-2^):

$F^{*}+F_{A}=F_{H}+F_{E}+F_{S}$, (SI-1)

where $F^{*}$ is the net radiation flux resulted from counteraction of solar and long-wave radiation fluxes, both of which consists of the upward and downward components; $F_{A}$ is positive (upward) energy flux due to anthropogenic heating of urban infrastructures; $F_{H}$ and $F_{E}$ are turbulent fluxes of sensible and latent heat; and $F_{S}$ is the net-storage heat flux. New advection is assumed to be negligible when compared to the other components.

The difference $\Delta F^{*}$ between the values of the net radiation flux in the non-polluted and polluted atmosphere leaves the anthropogenic heating unchanged, and causes variations ${\Delta F}_{H}$, $\Delta F_{E}$ and $\Delta F_{S}$ in all fluxes on the right hand side of Eq. (SI-1):

${\Delta F}^{*}={\Delta F}_{H}+\Delta F_{E}+\Delta F_{S}\approx{\Delta F}_{H}+\Delta F_{E}.$ (SI-2)

Here, the variation$\Delta F_{S}$ in the net-storage heat flux is neglected as small compared to the major variations: ${\Delta F}_{H}$ and/or $\Delta F_{E}$; and the sum ${\Delta F}_{H}+\Delta F_{E}$ represents the total turbulent heat-flux decrement caused by the air pollution. Clearly, the net radiation flux approaching the surface is smaller in the polluted atmosphere, so that ${\Delta F}^{*}<0.$

The major driving forth of the growth of convective BL is the near-surface value of the vertical turbulent flux of the buoyancy *b* defined as $b=\frac{g}{T_{0}}\theta+0.61gq$, where *g* is the acceleration due to gravity, $T_{0}$ is the reference value of absolute temperature, θ is the potential temperature, and *q* is specific humidity. The buoyancy flux is expressed through the heat fluxes $F_{H}$ and $F_{E}$:

$F_{b}=\frac{g}{c_{p}\varrho T_{0}}\left( F_{H}+\frac{0.61 c_{p}T_{0}}{L_{E}}F_{E} \right)\approx\frac{g}{c_{p}\varrho T_{0}}\left( F_{H}+0.07F_{E} \right)$, (SI-3)

where$\varrho$ is the air density, $c_{p}$ is the heat capacity at constant pressure, and $L_{E}$ is the latent heat of condensation.

As follows from (SI-2) and (SI-3), to a rough approximation the decrement in the buoyancy flux ${\Delta F}_{b}$ caused by the air pollution can be taken to be proportional to the easily measurable ${\Delta F}^{*}$.

**A1.2 Characteristic height of urban convective BL as dependent on the surface buoyancy flux**

A typical well-mixed urban convective BL grows from the city border along the wind, according to the prognostic equation:

$U\frac{dh}{dx}=a\frac{F_{b}}{N^{2}h}$, (SI-4)

where *h* is the BL height, *x* is the distance from the border, *U* is horizontal wind speed at the BL upper boundary, *a* is a dimensionless coefficient to be determined experimentally, and $N=\sqrt{\partial b/\partial z}$ is the Brunt-Väisälä frequency in the free troposphere *(37*). If no relevant data is available, *N* can be taken equal to its typical climatological value for given city and given month. A reasonable rough estimate is *N* $\approx$ 10^-2^ s^-1^.

The vertical turbulent flux of buoyancy $F_{b}=\bar{b'w'}$ is defined as the Reynolds-averaged product of fluctuations of vertical wind velocity ($w'$) and buoyancy ($b'$). In the CBL, $F_{b}$ is positive, which corresponds to the upward energy transport. In our analyses, this flux is determined directly from measurements of turbulent fluxes of potential temperature $\bar{w'\theta'}$ and specific humidity $\bar{w'q'}$; and it is demonstrated that $F_{b}$ essentially reduces in the polluted atmosphere.

Integrating Eq. (SI-4) yields:

$h=\sqrt{2a\frac{F_{b}x}{N^{2}U}}\approx\sqrt{2a\frac{F_{b}D}{N^{2}U}}$ , (SI-5)

where *D* is the horizontal city scale. The approximate relation (employing *D* as a characteristic distance) serves as a characteristic urban BL height. According to Eq. (SI-5), the ratio of the heights of the polluted and non-polluted urban convective BLs is equal to the square root of the ratio of the corresponding surface buoyancy fluxes:

$\frac{h_{poll}}{h_{nonpoll}}=\sqrt{\frac{F_{b,poll}}{F_{b,nonpoll}}}$ . (SI-6)

In windy weather, *U* is the speed of the background wind driven by the synoptic-scale pressure gradient. In calm weather, when the background wind is weak, the dominant role turns to the city-scale convective circulation cell driven by the urban heat island. Such cell produces its own convective wind estimated as

$U\sim A\left( F_{b}h \right)^{1/3}$, (SI-7)

where the combination $\left( F_{b}h \right)^{1/3}$ is the Deardorff convective velocity scale, and *A*$\sim$ *D/h* is the urban-cell aspect ratio that is essentially larger than unity. Then, substituting Eq. (SI-5) for *U* into Eq. (SI-6), yields the following alternative characteristic urban BL height:

$h\approx\left( 2a\frac{F_{b}^{2/3}D}{AN^{2}} \right)^{3/7}$ , (SI-8)

which implies a weaker than square root dependence: *h* ~$F_{b}^{2/7}$.

**A2 Correlation analysis**

Table 1 presents the correlation analysis for the radiation and flux parameters during the 8 month measurement period in Nanjing. The data presents cases when ambient RH is below 50% where the effect of hygroscopic water uptake of the aerosol particles remains small *(36)*.

**Supplementary Table 1.** Linear fits and associated correlation coefficients (*R*) between a) the aerosol mass (PM2.5) and b) BC concentration against the different radiation and flux components (y = *a_0_*x + *a_1_*). *N* is the number of data points.

|  |  | *a*_0_ | *a_1_* | *N* | *R* |
| --- | --- | --- | --- | --- | --- |
| a) PM_2.5_ | |  |  |  |  |
| *RH*<100% | *K_down_*/*K_top_* | -3.315⋅10^-4^ | 0.537 | 826 | -0.07 |
|  | *L_down_*/*K_top_* | 2.231⋅10^-4^ | 0.348 | 826 | 0.22 |
|  | *Q^*^*/*K_top_* | -4.929⋅10^-4^ | 0.385 | 826 | -0.15 |
|  | *F*_b_/*K_top_* | -1.594⋅10^-7^ | 4.889⋅10^-5^ | 767 | -0.34 |
| *RH*<80% | *K_down_*/*K_top_* | -3.049⋅10^-4^ | 0.5705 | 750 | -0.07 |
|  | *L_down_*/*K_top_* | 1.429⋅10^-4^ | 0.3529 | 750 | 0.13 |
|  | *Q^*^*/*K_top_* | -5.237⋅10^-4^ | 0.410 | 750 | -0.17 |
|  | *F*_b_/*K_top_* | -1.813⋅10^-7^ | 5.22⋅10^-5^ | 695 | -0.37 |
| *RH*<60% | *K_down_*/*K_top_* | -6.144⋅10^-4^ | 0.653 | 463 | -0.16 |
|  | *L_down_*/*K_top_* | 1.474⋅10^-4^ | 0.350 | 463 | 0.11 |
|  | *Q^*^*/*K_top_* | -7.441⋅10^-4^ | 0.460 | 463 | -0.27 |
|  | *F*_b_/*K_top_* | -2.087⋅10^-7^ | 5.699⋅10^-5^ | 432 | -0.36 |
| *b)* BC |  |  |  |  |  |
| *RH<100%* | *K_down_*/*K_top_* | -9.074⋅10^-3^ | 0.538 | 837 | -0.09 |
|  | *L_down_*/*K_top_* | 3.942⋅10^-3^ | 0.350 | 837 | 0.19 |
|  | *Q^*^*/*K_top_* | -11.495⋅10^-3^ | 0.385 | 837 | -0.17 |
|  | *F*_b_/*K_top_* | -3.164⋅10^-6^ | 4.84⋅10^-5^ | 777 | -0.33 |
| *RH<80%* | *K_down_*/*K_top_* | -8.268⋅10^-3^ | 0.573 | 754 | -0.09 |
|  | *L_down_*/*K_top_* | 2.981⋅10^-3^ | 0.353 | 754 | 0.14 |
|  | *Q^*^*/*K_top_* | -11.553⋅10^-3^ | 0.410 | 754 | -0.19 |
|  | *F*_b_/*K_top_* | -3.295⋅10^-6^ | 5.10⋅10^-5^ | 699 | -0.35 |
| *RH<60%* | *K_down_*/*K_top_* | -12.348⋅10^-3^ | 0.654 | 460 | -0.17 |
|  | *L_down_*/*K_top_* | 2.118⋅10^-3^ | 0.351 | 460 | 0.08 |
|  | *Q^*^*/*K_top_* | -15.709⋅10^-3^ | 0.461 | 460 | -0.29 |
|  | *F*_b_/*K_top_* | -3.962⋅10^-6^ | 5.63⋅10^-5^ | 429 | -0.35 |


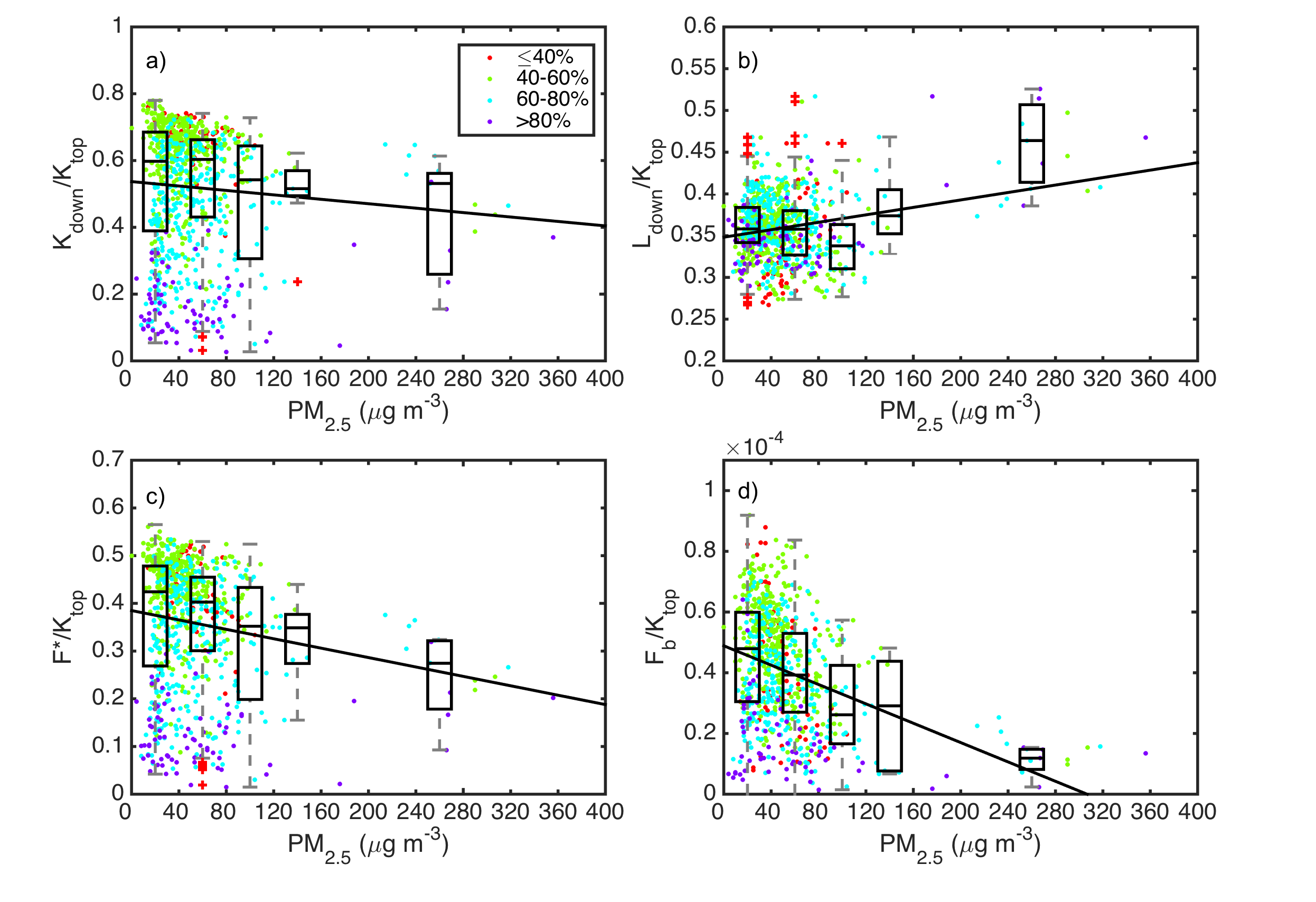


**Fig. A1.** Change in normalized a) solar radiation, b) longwave radiation, c) net all wavelength radiation and d) turbulent vertical flux of virtual temperature as a function of observed PM2.5 concentration. K_top_ is the solar radiation at the top of the atmosphere (W m^-2^). Only daytime conditions from the non-rainy periods between 10:00 and 14:00 local time are considered. The colour of the data points indicate observed relative humidity.

**
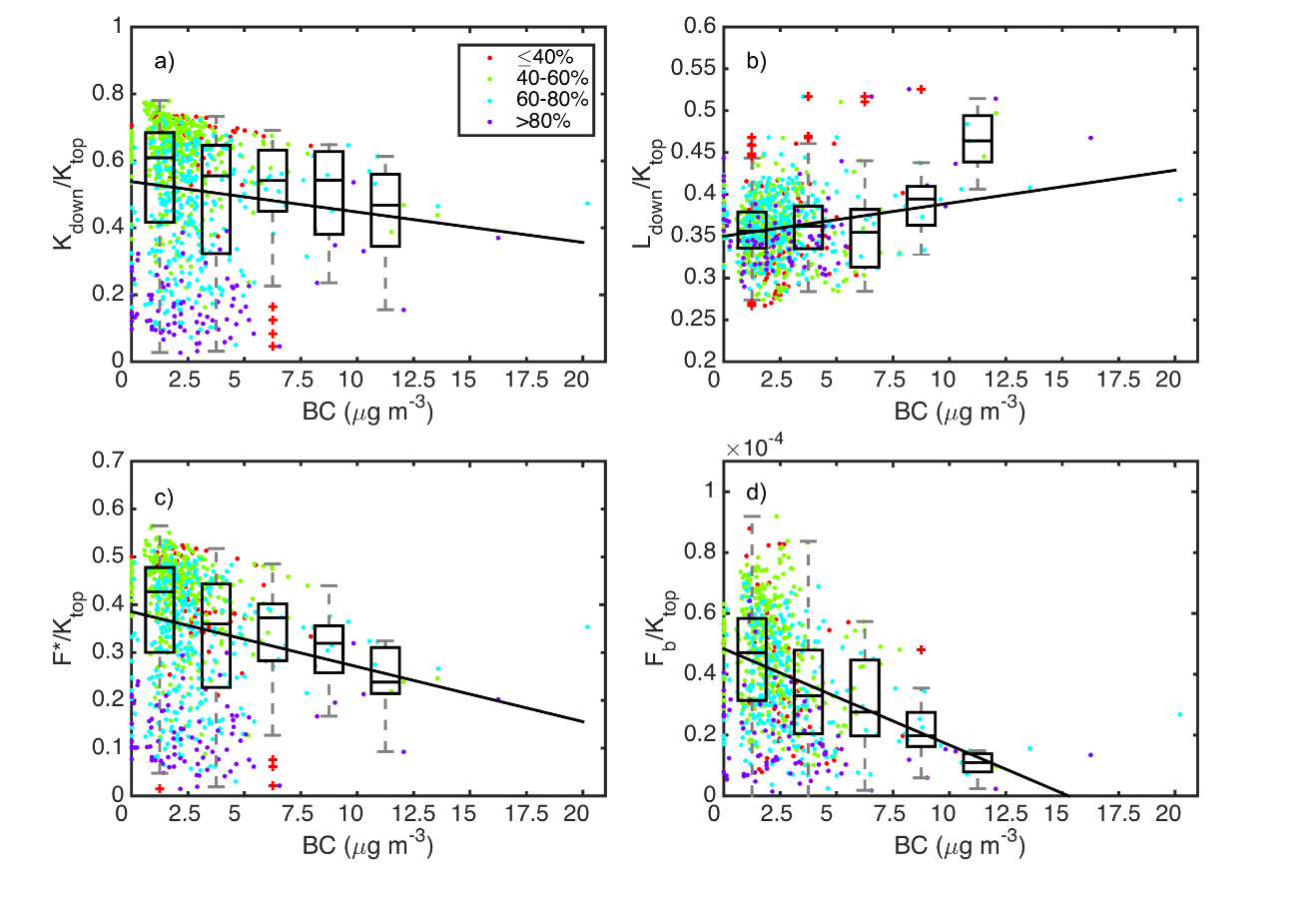
**

**Fig. A2.** A change in normalized a) solar radiation, b) longwave radiation, c) net all wavelength radiation and d) turbulent vertical flux of virtual temperature as a function of observed BC concentration. K_top_ is the solar radiation at the top of the atmosphere (W m^-2^). Only daytime conditions from the non-rainy periods between 10:00 and 14:00 local time are considered. The colour of the data points indicate observed relative humidity.
